# Myoblasts generated by lentiviral mediated MyoD transduction of myotonic dystrophy type 1 (DM1) fibroblasts can be used for assays of therapeutic molecules

**DOI:** 10.1186/1756-0500-4-490

**Published:** 2011-11-11

**Authors:** Jan Larsen, Olof J Pettersson, Maria Jakobsen, Rune Thomsen, Christina B Pedersen, Jens M Hertz, Niels Gregersen, Thomas J Corydon, Thomas G Jensen

**Affiliations:** 1 Department of Biomedicine, Aarhus University, Aarhus, Denmark; 2 Department of Molecular Medicine, Aarhus University Hospital, Skejby, Denmark; 3 Department of Clinical Genetics, Odense University Hospital, Odense, Denmark; 4 Research Unit for Molecular Medicine, Aarhus University Hospital, Skejby, Denmark

## Abstract

**Background:**

Myotonic dystrophy type 1 (DM1) is the most common muscle dystrophy in adults. The disease is caused by a triplet expansion in the 3'end of the myotonic dystrophy protein kinase (*DMPK) *gene. In order to develop a human cell model for investigation of possible effects of antisense and RNAi effector molecules we have used lentiviral mediated *myoD*-forced myogenesis of DM1 patient fibroblasts.

**Findings:**

Transduced fibroblasts show a multinuclear phenotype and express the differentiation marker myogenin. Furthermore, fluorescence in situ hybridization (FISH) analysis revealed a statistical significant increase in the amount of nuclear foci in DM1 patient fibroblasts after myogenesis. Finally, no nuclear foci were found after treatment with oligonucleotides targeting the repeat expansions.

**Conclusions:**

The abundance of nuclear foci in DM1 patient fibroblasts increase following myogenesis, as visualized by FISH analysis. Foci were eradicated after treatment with antisense oligonucleotides. Thus, we propose that the current cell model is suitable for testing of novel treatment modalities.

## Background

Myotonic dystrophy 1 (DM1) is a multisystemic dominant disease and it is the most common muscular dystrophy in adults [1]. The symptoms include muscle wasting (muscular dystrophy), cataract, heart conduction defects, insulin resistance, and myotonia. The current treatment is insufficient, ranging from muscle exercise to breathing assistance. The genetic cause of DM1 is a (CTG)n repeat in the 3'-untranslated region of the dystrophia myotonica protein kinase gene, *DMPK *[2] . Current evidence supports an RNA-gain-of-function pathogenesis [1]. Indeed, mutant *DMPK *mRNA localizes to distinct foci in the nucleus and sequesters multiple proteins, among these the alternative splicing regulator muscleblind-like protein 1 (MBNL1). This results in a depletion of MBNL1 in the nucleus, leading to multiple events of aberrant splicing. Other factors affected by the accumulation of foci include CUG-binding protein 1 (CUG-BP1) which is another alternative splicing regulator. Both MBNL1 and CUG-BP1 were recently shown to regulate the alternative splicing of numerous genes [3-5]. The importance of the nuclear foci has been underlined by the discovery that reduction of the number of foci is associated with normalized splice patterns in DM1 cells [6,7]. Foci abundancy and brightness has been reported to increase during myogenesis, but statistical analysis of the number of foci per cell was not performed [8].

To study DM1 pathogenesis in vitro, human DM1 myocytes can be used as model system. However, DM1 patient muscle cells are a scarce resource for research as a muscle biopsy is required to collect each sample. Transfer of the *myoD *gene has previously been shown to convert fibroblasts into myoblasts [9]. The myoD protein activates several transcription factors including myogenin. Inducible overexpression of *myoD *combined with a chloride channel luciferase minigene reporter system has been described recently for drug screening in a cell line [10]. Moreover, immortalized skin fibroblasts from a Duchenne Muscular Dystrophy patient have been used in a cell model, where cells were transduced with an inducible *myoD*-construct [11]. Here, we have characterized the reprogramming of DM1 patient fibroblasts to muscle cells by demonstrating a muscular phenotype and a statistical significant increase in the number of RNA foci per cell. Furthermore, we have used the cells for evaluating the treatment effect of previously described antisense oligonucleotides [7,12].

## Methods

### Cells and media

Normal human dermal fibroblasts (NHDF) were obtained from ATCC, USA. DM1 fibroblasts (GM03132) were from Coriell Institute, USA. Southern blotting analysis showed that the expanded allele contained approx. 2250 CTG repeats in the *DMPK *gene. Standard medium: DMEM from Invitrogen™ with 10% fetal calf serum (Sigma-Aldrich), glutamine, streptomycin and penicillin. Low serum medium (HS): F12 medium from Invitrogen™ containing 3% horse serum, glutamine, streptomycin and penicillin.

### Lentiviral production and transduction

The lentiviral vector encoding *myoD *was generated by replacing the *puro *gene in pCCL-WPS-PGK-puro-WHV [13] with *myoD *cDNA. For lentiviral production, 293T cells were seeded at 3 × 10^6 ^cells/p10 dish in standard medium, which was refreshed one hour prior to transfection. Cells were transfected by a CaPO_4 _co-precipitation method with 3.75 μg *pMD.2G*, 3 μg *pRSV-Rev*, 13 μg *pMDGP-Lg/RRE *and 13 μg of transfer vector (either *pCCL-WPS-PGK-MyoD-WHV *or *pCCL-WPS-PGK-GFP-WHV*). The medium was refreshed 24 hours post-transfection. One day later, supernatant containing the viral vector was filtered through a 0.45 μm pore filter, and polybrene added to a final concentration of 8 μg/ml. The medium was diluted 1:3 with standard medium and transferred to NHDF and DM1 fibroblasts. Medium was refreshed 24 hours after transduction.

### Quantitative RT-PCR

RNA was isolated and cDNA was synthesized according to manufacturer's protocol (Sigma^® ^and BioRad^®^, respectively). Before use, the cDNA was thawed and diluted appropriately; in this range of experiments a dilution of 1:4 was used. Furthermore, dilutions were made to set up a standard curve for the reactions. 2 μl of cDNA 1:4 dilution of each sample was added to 23 μl solution consisting of 12.5 μl TaqMan^® ^universal PCR mastermix, 1.25 μl TaqMan mRNA specific primer set and 9.25 μl H_2_O. Reaction plates were analyzed by an ABI Prism® 7000 sequence detection system (Applied Biosystems). All samples were analyzed as triple determinations. The following TaqMan^® ^assays were used: *myogenin *(MYOG, Hs01072232_m1, Applied Biosystems) and *myoD *(MYOD1, Hs00159528_m1, Applied Biosystems).

### Immunofluorescence (IF)

Human primary fibroblasts were grown in either standard medium or F12 with 3% horse serum (HS medium) at 37°C in 5% CO_2_. The cells were grown to appropriate confluence and fixed in 4% formaldehyde (Lilly's solution) for 10 minutes. After fixation, the cells were washed 3 times in PBS (137 mM NaCl, 2.7 mM KCl, 10 mM Na_2_HPO_4_, 2 mM NaH_2_PO_4 _(pH 7.2)). To lower the non-specific binding, the slide was incubated in 100 μl 1% BSA in PBS (blocking buffer) prior to antibody addition. Slides were washed in PBS and incubated with 100 μl primary antibody diluted (1:100) in blocking buffer (BSA-PBS) for 2 hours. 3× wash in PBS followed before 100 μl secondary antibody (1:400) diluted in blocking buffer, was added. The slide was incubated with secondary antibody for 1 hour before it was washed and 100 μl antifade, with or without DAPI (4',6-diamidino-2-phenylindole) was added. Primary antibodies were mouse monoclonal anti-myoD and anti-myogenin (both Santa Cruz Biotechnology). The secondary antibodies were Alexa Flour^® ^488 conjugated goat anti mouse (Invitrogen). All secondary antibodies were diluted 1:400 in 1% BSA PBS solution.

### Fluorescence in situ hybridization (FISH)

FISH was performed according to protocol from SingerLab Online [14].

Probes were RP-HPLC purified and labeled with Cy3 (red) purchased from DNA Technology A/S, Denmark. The Cy3-labeled probe consisted of a (CAG)_10_-sequence with a fluorophore at the 5'-end [15]. All reagents used in the above protocol were nuclease free and dissolved/diluted in nuclease free/DEPC-treated water or PBS unless otherwise stated. The numbers of RNA foci were counted microscopically and compared using Student's t-test.

### Quantification of foci

The numbers of foci per cell were quantified by two different methods giving essentially similar results. The first method was based on direct counting, using a fluorescence microscope. Microscope fields were chosen randomly, using the DAPI filter, and dots were counted in all cells where the complete cell was present in the field. In another method images were acquired. This was done (in the Cy3 channel), using multidimensional acquisition, and images were stacked into a 2d-picture. The counting was performed by allowing the software to count the number of foci based on an arbitrary threshold value for minimum light intensity. Software used in the image acquisition was MetaMorph, and in the image processing, including counting, ImageJ was used.

### Combined IF and FISH

Cells were rehydrated and permeabilized according to the FISH procedure described above. Next, cells were blocked in 1%BSA solution in a humidified hybridization chamber for 1 hour at room temperature. Afterwards, cells were transferred to a new hybridization chamber and incubated with primary antibodies for 1.5 hours at 37°C. Following this, cells were washed three times 5 minutes in PBS containing 5 mM MgCl_2 _at room temperature and thereafter incubated in secondary antibodies for 45 minutes at 37°C in a humidified hybridization chamber. Subsequently, cells were washed five times 5 minutes in PBS containing 5 mM MgCl_2 _at room temperature. Cells were fixed in 4% paraformaldehyde for 20 min at room temperature and the FISH procedure was performed as described above.

### Transfection with RNA oligonucleotides

Oligonucleotides used in the present study comprised 21 nucleotides. They were chemically modified, as previously described [7] with a phosphorothioate backbone (PT) and methylated at 2'O (2'OMe). The antisense oligonucleotide had the sequence (CAG)7, targeting the pathogenic repeat. The control oligonucleotide had the following sequence: GUAGCGACUAAACACAUCAAG.

Cells were transferred to glass cover slips in a 12-well plate with a confluency of 1.5 × 10^5 ^cells/well. The following day, cells were transfected with the oligonucleotide, using polyethyleneimine (PEI) aided by hyaluronic acid (HA) as transfection reagent (Sigma-Aldrich). Transfection was performed as previously described [16].

Oligonucleotides (600 μg/ml), hyaluronic acid (HA) (Sigma-Aldrich; 3.5 mg/ml) and polyethyleneimine (PEI) (Sigma-Aldrich; 937.5 μg/ml) were mixed in a 1:2:1 ratio (by volume). This suspension was diluted in an equal volume of doubly concentrated PBS. The DNA/HA/PEI/PBS solution was incubated for 30 minutes at room temperature. Immediately before adding the complex to the cells, the medium was refreshed (ordinary DMEM) and the complex was added to the cells. Cells were incubated for 4 hours at 37°C with the transfection complex and the medium was subsequently replaced with fresh DMEM. Cells were fixed for FISH analysis 24 hours post-transfection.

## Results and discussion

### Analysis of *myoD*-transduced primary human fibroblasts

**Figure 1:**
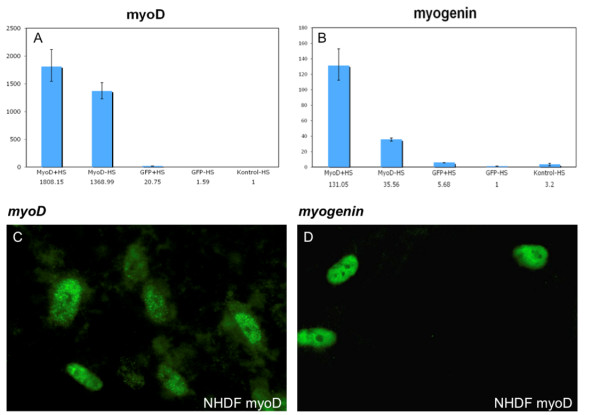
**Expression of *myoD *and *myogenin *in normal human dermal fibroblasts (NHDF)**. (A and B) The cells were cultured for 6 days before harvest for quantitative RT-PCR or (C and D) IF: *myoD *and *myogenin *expression relative to *beta-actin*. +HS indicate cells grown in low serum medium; -HS designates cells grown in standard DMEM medium. Control designates no transduction. (C and D) Immunofluorescence of myoD and myogenin proteins, respectively. Original magnification × 630.

Lentiviral mediated gene transfer was used for expression of myoD in normal human dermal fibroblasts (NHDF). Quantitative PCR (qPCR) analysis was used to determine the expression of *myoD *and *myogenin *in transduced NHDFs. Cell differentiation was enhanced using low serum medium (HS) containing 3% horse serum (see materials and methods) [17]. We observed that *myoD *was expressed in fibroblasts 6 days after lentiviral transduction (Figure 1A). Cells transduced with *GFP *and non-transduced cells expressed negligible quantities of *myoD *relative to cells transduced with *myoD*. The levels of *myoD *RNA were further increased in the *myoD*-transduced cells cultured in HS medium. Analysis by qPCR likewise showed that the differentiation marker *myogenin *is expressed in cells transduced with *myoD*, and further increased by cultivation in HS medium (Figure 1B). Immunofluorescence revealed that both myoD and myogenin could be detected in *myoD*-transduced fibroblasts (Figure 1C, D). As expected, both proteins were located in the nuclei. Neither myoD nor myogenin were observed in non-transduced or *GFP-*transduced cells (data not shown).

### *myoD*-transduction of DM1 fibroblasts leads to a muscle-like phenotype and increased numbers of nuclear RNA foci

**Figure 2:**
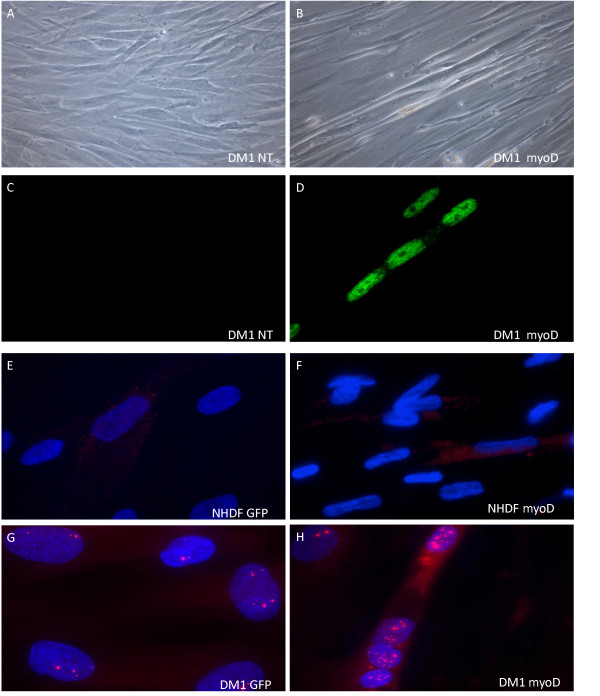
**Characterization of DM1 patient fibroblasts (PT) transduced with *myoD***. (A and B) Light microscopy of PT cells cultured in low serum medium for 21 days post-transduction. (A) Non-transduced (NT) PT cells display a fibroblast phenotype, (B) PT cells transduced with *myoD *display elongated tubular morphology. (D) IF of myogenin in DM1 patient fibroblasts (PT), 12-days post-transduction PT cells express myogenin and exhibit multinuclear morphology, (C) while non-transduced PT cells do not. (E-H) *myoD *induced myogenesis increase the number of nuclear foci in DM1 patient fibroblasts. (G-H) DM1 patient fibroblasts and (E-F) normal fibroblasts were cultured 10 days in low serum medium following transduction with (E and G) *GFP *or (F and H) *myoD*. Cells were analyzed by FISH with a Cy3-labeled (CAG)_10 _probe (red) targeting the pathogenic CUG repeat expansion of DMPK mRNA. (E-F) Normal fibroblasts (NHDF) displayed no nuclear foci when transduced with *GFP *or *myoD*. (G-H) DM1 patient fibroblasts exhibited an increased number of nuclear foci in fibroblasts transduced with *myoD *relative to *GFP *transduced fibroblasts. (H) The high number of foci was only observed in *myoD *transduced fibroblasts with myogenic morphology. (A-B) Original magnification × 400 and (C-H) × 630.

**Figure 3:**
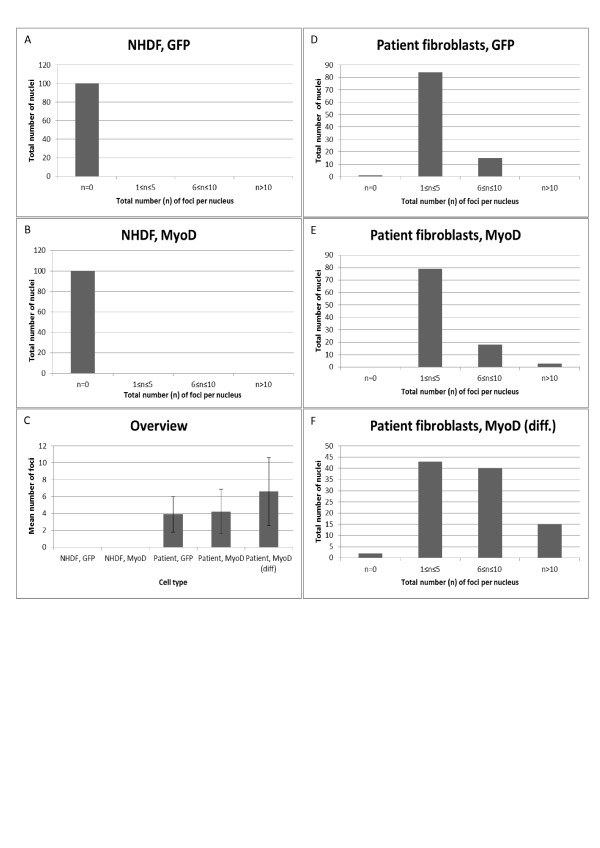
**Quantification of foci in DM1 patient cells and NHDF**. (A and D) Fibroblasts were transduced with *GFP *or (B, E-F) *myoD*. Nuclear foci in 100 cell nuclei were counted for each sample. (A and B) NHDF cell nuclei contained no foci following tranduction with *GFP *or *myoD*. (D and E) PT cells transduced with *GFP *or *myoD *showed foci in cell nuclei. (F) Differentiated (*myoD*) patient fibroblasts displayed markedly changed morphology and exhibited an increase in the average as well as the maximum number of nuclear foci per cell.

Having shown myogenesis of normal cells, *myoD*-transduced DM1 fibroblasts were cultivated in low-serum medium (HS) for 2-3 weeks. Cellular differentiation was mediated by lentiviral delivery of the transcription factor *myoD*. As seen in Figure 2, *myoD-*transduced cells display multinuclear tubular morphology and express myogenin (Figure 2B, D), respectively, compared to non-transduced cells (Figure 2A, B). Fluorescence in situ hybridization (FISH) using a Cy3-labeled (CAG)_10 _oligonucleotide probe revealed distinct nuclear foci in DM1 patient fibroblasts (Figure 2G and 2H), whereas no foci were found in NHDF (Figure 2E, F). Statistical analysis of the number of foci in *myoD*-transduced DM1 cells revealed that transduction with *myoD *lead to increased numbers of Cy3-foci per nucleus (Figure 3). The increased foci abundancy was only seen in myoD-transduced cells with a myogenic phenotype (p < 0.01; Student's t-test) (Figure 3F).

**Figure 4:**
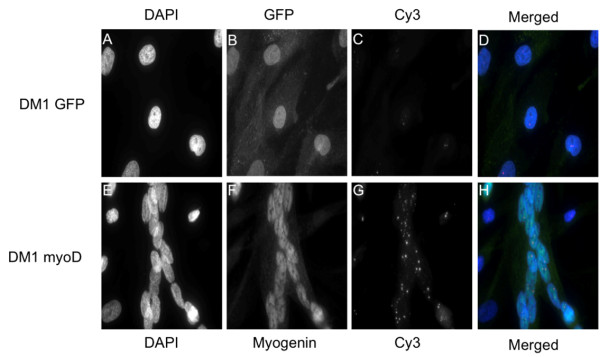
**IF/FISH targeting myogenin (IF) and the (CAG)-repeat (FISH) of transduced DM1 fibroblasts**. (A and E) Panels display DAPI counterstain, (B and F) FITC/GFP channel, (C and G) Cy3 channel, (D and H) merge of DAPI/GFP/Cy3. (A-D) Cells transduced with *GFP *display no change in morphology or in the number of Cy3-foci (red) top panel. (E-H) Cells transduced with *myoD *display a multinuclear morphology, signs of cell fusion (syncytia), and an increased number of Cy3-foci per nucleus. Primary antibodies target myogenin, secondary antibodies are Alexa Fluor 488-labeled. The probe is a Cy3-labeled (CAG)_10 _DNA oligonucleotide. Original magnification × 630.

Combined immunostaining and fluorescence in situ hybridization (IF/FISH) revealed that only cells transduced with *myoD *had multiple nuclei, tubular morphology and an increased number of nuclear foci (Figure 4E-H). Furthermore, myogenin was expressed in all nuclei of cells with multiple nuclei and tubular morphology (Figure 4F, H). Cell fusion was only found between cells in which all nuclei expressed myogenin (Figure 4F, H). The morphology of *myoD-*transduced normal fibroblasts was similar to that observed in *myoD*-transduced DM1 patient fibroblasts. Thus, combined immunostaining and fluorescence in situ hybridization confirmed that myogenesis leads to significantly increased numbers of nuclear RNA foci per cell.

### *myoD*-transduced DM1 patient fibroblasts can be used for analysis of treatment effects of antisense oligonucleotides

**Figure 5:**
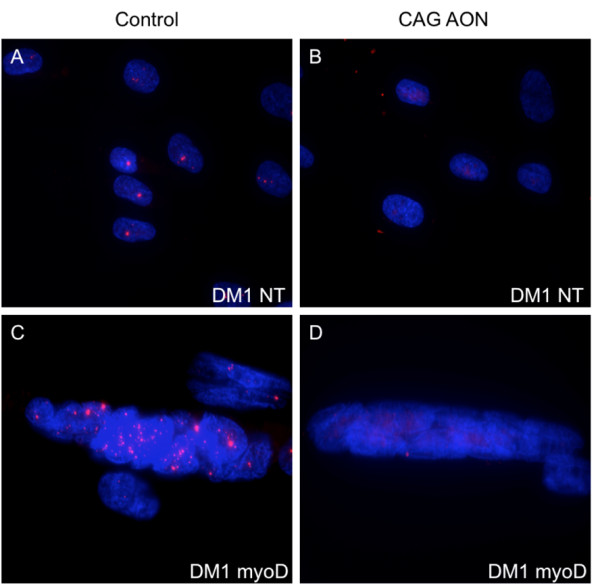
**Treatment of DM1 cells with antisense oligonucleotides**. Cells were transfected with 1 μg of oligonucleotides (AON) targeting the CUG triplet repeat, and RNA foci visualized using FISH with a Cy3-labeled CAG probe (red dots). (C and D) Differentiated DM1 cells cultivated in HS medium, 2 weeks post-*myoD*-transduction. (A and B) DM1 fibroblasts. (B and D) Cells transfected with AONs. (A and C) Untransfected. Original magnification × 630.

DM1 patient cells were transfected with antisense oligonucleotides (AONs) targeting the CUG repeat in *dmpk *mRNA. Already one day after treatment, FISH revealed elimination of the nuclear foci, both in non-transduced and *myoD-*transduced cells transfected with the AONs (Figure 5B, D). Transfection with control oligonucleotides with the same length and chemical modifications did not affect the number of foci (data not shown). It has previously been shown that the present AONs reduce the levels of mutant DMPK RNA [7]. However, although evaluation of treatment effects 2 days after AON transfection gave similar results (data not shown), it cannot be completely ruled out that the observed eradication of foci could be a consequence of competition between the therapeutic AONs and the labeled FISH probe.

## Conclusions

In DM1 muscle cells the transcription factor *myoD *is downregulated, and poor muscle differentiation has been described [18,19]. However, normal myogenin expression has been shown in developing myoblasts isolated from DM1 patient biopsies [20]. We here show that lentiviral mediated *myoD*-transduction leads to *myoD *and *myogenin *expression in both normal and DM1 fibroblasts, indicating that the impaired differentiation can be overcome at least to some extent. Myogenin regulates myotube formation and accordingly we detected myogenin in all nuclei of cells with a myotubular phenotype (Figure 4H).

Foci of nuclear mutant *DMPK *is a characteristic manifestation of the DM1 disease phenotype. These foci were visualized by FISH analysis, using a Cy3-labeled CAG-probe [15,20-25]. The appearance of the foci differed considerably, both in shape, intensity and area (size). As previously described, the vast majority were localized to the nucleus and the intranuclear position was seemingly random [15]. We observed an increase in the number of foci in differentiated cells, displaying a mature muscle phenotype (Figure 3). This is in agreement with earlier reports using retrovirally transduced DM1 fibroblasts [8]. Both NHDF and DM1 patient cells were found to be responsive to *myoD*-forced myogenesis (Figure 1 and 2) as has been reported previously [8-10,25]. However, the myogenic capability and increase in foci abundancy is slightly controversial, since earlier reports indicate decreased differentiation in DM1 cells [18,19]. In contrast, a more recent study reported normal myogenesis, but increased apoptosis in DM1 cells [20]. Indeed, we observed normal myogenesis in the DM1 cells, and no obvious signs of increased cell death were seen in the differentiated DM1 fibroblasts compared to the differentiated NHDF fibroblasts. Transfection with antisense oligonucleotides (AONs) can lead to reversal of RNA toxicity in a DM1 cell model from transgenic mice through elimination of RNA foci [7]. To ascertain that differentiated DM1 cells are applicable as a human cell model, we reproduced foci reduction in differentiated human DM1 cells (Figure 5), by transfecting with antisense oligonucleotides previously shown to be capable of rescuing the phenotype [12]. Thus, *myoD*-transduced DM1 cells are capable of myogenic differentiation and can be used as a cell model for in vitro experiments studying the cellular pathophysiology and possible effects of therapeutic compounds on the DM1 phenotype.

## Competing interests

The authors declare that they have no competing interests.

## Authors' contributions

JL and OJP contributed equally to this work, carried out the molecular genetic studies and drafted the manuscript. MJ constructed the lentiviral vector. RT consulted on the immunoassays and in situ hybridizations. CBP and NG consulted on the RTQ-PCR assay. JMH and TJC participated in study design and helped to draft the manuscript. TGJ conceived of the study, and participated in its design and coordination and helped to draft the manuscript. All authors read and approved the final manuscript.
